# The Influence of Physical Exercise on Adolescent Personality Traits: The Mediating Role of Peer Relationship and the Moderating Role of Parent–Child Relationship

**DOI:** 10.3389/fpsyg.2022.889758

**Published:** 2022-06-10

**Authors:** Yi Liao, Xiaoyu Cheng, Wei Chen, Xiaowei Peng

**Affiliations:** College of Physical Education, Wuhan Sports University, Wuhan, China

**Keywords:** physical exercise, adolescent, personality traits, peer relationship, parent-child relationship

## Abstract

Adolescence is the critical period of the formation for individual personality traits, which would be influenced by numerous factors such as the internal and external environment. In view of physical exercise as an important factor affecting the healthy development of adolescents, whether it would play an important role in the formation of adolescents’ personality traits and how it would work deserve further investigation. Based on the Ecological Systems Theory, this study has explored the relationship between physical exercise and adolescents’ personality traits, as well as the mediating effect of peer relationship and the moderating effect of parent–child relationship using 9,284 data samples. The regression results show that physical exercise has a significant positive impact on the development of personality traits such as neuroticism, conscientiousness, and agreeableness. Peer relationships exert the mediating effect between physical exercise and adolescents’ personality traits. However, parent–child relationship only moderates the effect of physical exercise on conscientiousness and agreeableness.

## Introduction

Personality trait is an inherent individual neuropsychological structure (McCrae and Costa, 1997), which refers to an individual’s behavior tendency in a relatively consistent way at different times and in different situations (Simon et al., 2020). Personality trait could effectively predict individual behaviors, which is an important characteristic that distinguishes individuals from others. Since the 1980s, Five-Factor Model (FFM) has been largely accepted and adopted as a theoretical model of personality traits, that is, neuroticism, extroversion, openness, agreeableness, and conscientiousness (Hendriks et al., 1999). Among them, neuroticism reflects the degree of individual emotional stability and the behavioral tendencies toward negative emotions (Laursen et al., 2010); extroversion reflects the interaction degree of individual interpersonal communication (Mesurado et al., 2014); openness reflects an individual’s own creativity and curiosity (Gjerde and Cardilla, 2009); agreeableness reflects the individual’s attitude in the process of getting along with others (Laursen et al., 2010); conscientiousness reflects the individual’s prudence and persistence degree in goal-directed behaviors (De Fruyt et al., 2017). Studies have shown that compared with adults, adolescents’ personality traits tend to show greater volatility and plasticity and be more susceptible to be influenced by the external environment such as family, parent–child relationship, and peer relationship (Roberts and DelVecchio, 2000; Roberts et al., 2006; Fishman et al., 2011; Childs et al., 2014; Tottenham and Galván, 2016).

Physical exercise, an important factor influencing adolescent’s development, has attracted more and more attention of scholars to identify the effect on the development of adolescents’ personality traits. It has been found that physical exercise could affect the development of personality traits (Allen and Laborde, 2014; De Fruyt and Van Leeuwen, 2014; Wilson and Dishman, 2015). Some studies have shown that participation in physical exercise has a positive impact on the development of individual personality traits (Kwon, 2018), which could help the individual to improve the stability of personality traits. For adolescents, physical exercise is an important way for social communication, and active participation in physical exercise could help them improve extroversion and agreeableness. Moreover, when individuals take part in physical exercise, complying with the certain required discipline and the sportsmanship of cooperation, regulations, fairness, fortitude, and so on (Eime et al., 2013; Stephan et al., 2014b) could effectively improve adolescents’ self-confidence, self-esteem, sense of responsibility, self-management ability, and emotional regulation ability, as well as the stability of personality trait (Vukasović Hlupić and Bratko, 2015; Alkadhi, 2018). Based on the above analysis, hypothesis 1 has been proposed:

Hypothesis 1: Physical exercise has a significant impact on adolescents’ personality traits.
Hypothesis 1a: Physical exercise has a significant impact on adolescents’ neuroticism.
Hypothesis 1b: Physical exercise has a significant impact on adolescents’ conscientiousness.
Hypothesis 1c: Physical exercise has a significant impact on adolescents’ agreeableness.

What needs to be further discussed is the influencing mechanism between physical exercise and adolescents’ personality traits. The existing studies have mainly used psychological mechanisms, such as self-efficacy and resilience, as mediating or moderating variables to explore the influencing mechanism. The social mechanism has seldom been discussed. However, Bronfenbrenner’s Ecological Systems Theory (EST) has identified that personal growth is a complicated process, which is affected by many factors such as social factors and the environmental systems (Bronfenbrenner, 1979). In the process of personal growth, individuals would be connected closely with ecosystems and be affected by various ways. In EST, Bronfenbrenner has divided ecosystems into five categories, namely, microsystems, meso-systems, exosystems, macrosystems, and chronosystems (Bronfenbrenner, 1979). Among them, microsystems refer to the systems that individuals are directly exposed to in their social life, such as the family, peers, and school, which would exert an imperceptible influence on their behavioral styles and values. In the development of adolescents’ personality traits, family and peers are two important microsystems with the development of individuals’ personality traits. Peer and parent–child relationships have a positive effect in the development of adolescent’s personality traits (Caires et al., 2009).

First, peer relationship plays an important role in physical exercise and the adolescent’s personality traits (Koepke and Denissen, 2012; Klimstra et al., 2013). With the development of adolescents’ independence and autonomy, the peer relationship plays a more irreplaceable role in adolescents’ socialization and emotional development (Gorrese, 2015). The existing studies have shown that participation in physical exercise could effectively promote the development of individual peer relations, expand the individual’s range of interpersonal interactions, and increase the number of friends (Smith, 2003; Jowett and Lavallee, 2007; Eime et al., 2013; Swanson et al., 2019). In addition, the studies have identified that peer relationship exerts the mediating effect in the relationship between physical exercise and output variables. Participation in physical exercise could further increase the levels of individuals’ extraversion, agreeableness, and responsibility through peer effects (Konu et al., 2002; Lehto et al., 2012). Second, for the parent–child relationship, the attachment theory by Bowlby has stated that individuals would seek attachment objects to prevent danger and improve chances of survival by establishing emotional connection (Bowlby, 1969). In the process of interaction with parents, individuals could observe the sense of security, love, and trust, and learn to understand others. Parents are the important attachment figures for adolescents. The relationship between adolescents and parents is crucial in shaping adolescents’ personality traits. Moreover, a good parent–child relationship plays an “incubator” role in the development of adolescents’ personality traits (Davison, 2004; Craig et al., 2013), which could provide emotional support and make them more brave and confident in making friends (Freedson and Evenson, 1991; Davies and Cummings, 1994). On the contrary, low-quality or poor parent–child relationship would weaken the positive effect or even cause the negative effect on the development of personality traits (Strong et al., 2005; Kılınç and Sis Çelik, 2021). In addition, appropriate intervention of parents could effectively weaken the negative influence of peer on adolescents. Based on these facts, the study puts forward hypothesis 2 and hypothesis 3:

Hypothesis 2: Peer relationship has a mediating effect on the relationship between physical exercise and adolescents’ personality traits.
Hypothesis 3: Parent–child relationship has a moderating effect on physical exercise and adolescents’ personality traits. The direct effect of physical exercise and peer relationship on adolescents’ personality traits is moderated by parent–child relationship.

**FIGURE 1:**
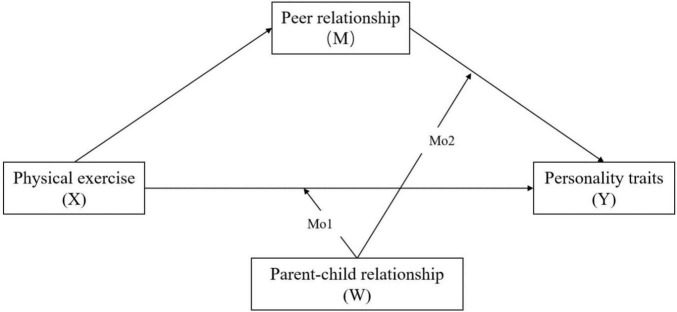
Conceptual framework of the proposed moderated mediation model.

Based on the above analysis, the theoretical model of the physical exercise on the adolescent’s personality traits has been explored (see Figure 1), in order to identify the relationship between physical exercise and adolescents’ personality traits as well as the mediating role of peer relationship and moderating role of parent–child relationship based on the 9,284 adolescent’s data samples. According to the EST, this study aims to further reveal the influencing mechanism of adolescent’s personality traits from the perspective of social relationship, and provide the theoretical support and practical guidance for promoting adolescent’s physical exercise and personality trait development.

## Materials and Methods

### Material Source

The data were collected from the China Education Panel Survey (CEPS), which was designed and implemented by the National Survey Research Center (NSRC). This project took the seventh and ninth-grade middle school students in the 2013–2014 academic year as the baseline survey respondents through the probability proportionate to size sampling (PPS) method and 19,487 students from 438 classes in 112 schools in China have been investigated. In the follow-up survey in the academic year 2014–2015, 9,449 respondents in the first-round baseline survey have been monitored. The survey data were nationally representative and covered multi-dimensional information related to students, families, schools, and other fields. A total of 9,284 samples has been finally obtained by combining the first-round and follow-up survey data through excluding the data with missing values. The characteristics of selecting variables in this study have been summarized in Table 1.

**TABLE 1:** Sample descriptive statistics information (*N* = 9,284).

	Variable	Mean/Ratio	S.D.	Min	Max
Independent variable	Physical exercise	0.37	0.40	0.00	5.00
Dependent variable	Neuroticism (time 1)	4.20	0.88	0.00	5.00
	Conscientiousness (time 1)	3.25	0.69	0.00	4.00
	Agreeableness (time 1)	3.50	0.71	0.00	4.00
	Neuroticism (time 2)	4.80^a^	0.81	0.00	5.00
	Conscientiousness (time 2)	3.67^a^	0.64	0.00	4.00
	Agreeableness (time 2)	3.49^b^	0.68	0.00	4.00
Control variables	Gender	0.52	0.45	0.00	1.00
	National	0.92	0.27	0.00	1.00
	Registered residence	0.52	0.50	0.00	1.00
	Health	0.58	0.49	0.00	1.00
	Cognitive ability	0.50	0.87	–2.03	2.33
	One child/Only child in family	0.45	0.49	0.00	1.00
	Parental education level	2.57	0.94	0.00	5.00
	Family cultural capital	3.25	1.22	1.00	5.00
	Family economic	0.79	0.41	0.00	1.00
	School infrastructure	2.08	0.40	1.20	3.00
	School type	0.94	0.24	0.00	1.00
	School ranking (Above average)	0.59	0.49	0.00	1.00
	School ranking (The best)	0.23	0.42	0.00	1.00
	School location (A combination of urban and rural areas)	0.26	0.44	0.00	1.00
	School location (City center)	0.34	0.47	0.00	1.00
Mediating variable	Peer relationship(time 1)	2.76	0.28	1.00	3.00
Moderating variable	Parent-child relationship(time 1)	2.10	0.47	0.00	3.33

*^a^Significantly lower than the baseline value. ^b^Significantly higher than the baseline value.*

### Physical Exercise

The independent variable of the study was physical exercise. In the baseline survey, the two items of *“How long do you exercise on average from Monday to Friday,” “How long do you exercise on weekends”* have been designed in CEPS to examine the situation of participation in physical exercise. The calculation of the duration time of physical exercise was “Exercise time = (physical exercise time from Monday to Friday + physical exercise time on weekends) /60.”

### Personality Traits

The dependent variable was personality traits. Variables have been measured by five dimensions in the CEPS questionnaire, which are neuroticism, extroversion, openness, agreeableness, and conscientiousness. The neuroticism dimension was measured by five items (Wang et al., 2020; Zhao and Chen, 2022), that is, *“you often feel “blue,” “depressed,” “unhappy,” “not enjoying life,” and “sad” in the past seven days,”* using five-point Likert scale ranging from 1 (never) to 5 (always), in which the Cronbach’s α is 0.904. The extraversion dimension was measured by four items, that is, *“there are some adults I respect and admire” “I can easily talk to adults” “I apologize when I accidentally hurt or offend people” “If I handle things, the method is wrong and I will try to find another way to solve it,”* using four-point Likert scale ranging from 1 (completely agree) to 4 (completely disagree), in which the Cronbach’s α is 0.680. The openness dimension was measured by 4 items, that is, *“I can express my opinions clearly” “I can respond quickly” “I can learn new knowledge quickly” “I am curious about new things,”* using four-point Likert scale ranging from 1 (completely agree) to 4 (completely disagree), in which the Cronbach’s α is 0.714. The agreeableness dimension was measured by four items, that is, *“Most of my classmates are nice to me” “My class is in good atmosphere” “I often take part in school/class activities” “I feel close to people in this school,”* using four-point Likert scale ranging from 1 (completely agree) to 4 (completely disagree), in which the Cronbach’s α is 0.773. The conscientiousness dimension was measured by four items, that is *“I would try my best to go to school even if I had any reasons to stay at home” “I would try my best to finish even the homework I dislike” “I would try my best to finish my homework, even if it would take me quite a long” “I would persist in my interests and hobbies,”* using four-point Likert scale ranging from 1 (completely agree) to 4 (completely disagree), in which the Cronbach’s α is 0.805. The whole Cronbach’s α is 0.814.

Personality traits are the unique psychological characteristics of individuals formed in the long-term living environment. Although they often show greater volatility and plasticity in adolescence, the impact of physical exercise on adolescents’ personality traits may still take some time to have an impact. Therefore, to better illustrate these changes of personality traits, the longitudinal analysis to measure adolescent personality traits(Δt) based on the tracking data of personality traits in CEPS has been conducted. The specific calculation method is as follows: Δt = score t2-score t1 (where score t2 is the score on each dimension of adolescent personality traits in the first follow-up survey; score t1 is the score on each dimension of adolescent personality traits in the baseline follow-up survey). Considering the unreliability of difference scores, the overall variable of personality traits and a new regression model as the robustness test of regression analysis has been conducted (Stephan et al., 2014b). Due to the data acquisition of CEPS, the dimensions of neuroticism, conscientiousness, and humanity have been taken as an example.

### Peer Relationships

The mediating variable was peer relationship. In the baseline survey in CEPS, this variable was measured by ten items about adolescent’s peer relationship, that is, *“Do any of these things happen to your best friends: “Doing well in academic performance “Studying hard” “Expecting to go to college” “Skipping classes” “Criticized or punished for violating school rules” “Always fighting with others” “Smoking or drinking alcohol” “Always going to net bars or video arcade” “Having had or is having a romance” “Dropped out of school,””* using three-point Likert scale ranging from 1 (no) to 3 (many), in which the Cronbach’s α is 0.831. The scores of items on negative peer influence were calculated in a reverse order. The whole score of peer relationship was calculated by averaging the values of the ten dimensions.

### Parent–Child Relationship

The moderating variable was parent–child relationship. Three dimensions of parent–child communication, parent–child interaction, and parent–child trust were selected to measure the parent–child relationship (Zheng et al., 2020; Liu and Jiang, 2021). In the baseline survey in CEPS, parent–child communication was measured by ten items, that is, *“Do your mother (father) often discuss the following issues with you?: “Things happened at school” “The relationship between you and your friends” “The relationship between you and your teachers” “Your feelings” “Your worries and troubles,””* using three-point Likert scale ranging from 1 (never), 2 (sometimes) to 3 (often), in which the Cronbach’s α is 0.868. The score was obtained by summing and averaging the 10 items with a value in the range of 1–3. Parent–child interaction was measured by six items in CEPS, that is, *“The frequency with which you and your parents do the following is approximately: “Having dinner” “Reading” “Watching TV” “Playing sports” “Visiting museums, zoos, science museums, etc.” “Going out to watch movies, shows, sports games, etc.,””* using six-point Likert scale ranging from 1 to 6 (never, once a year, once every half year, once a month, once a week, more than once a week), in which the Cronbach’s α is 0.773. The score was obtained by summing and averaging the six items with a value in the range of 1–3. Parent–child trust was measured by three items in CEPS, that is, *“Who will be the first one for you to turn to when you want to chat with someone?” “Who will be the first one for you to turn to when you are in trouble?” “Who will be the first one for you to turn to when you need help?”* in which the Cronbach’s α is 0.704. The “parent” option was assigned to 1 and the other options to 0. After the virtualization processing, the score was obtained by summing and averaging the three items with a value in the range of 0–1. Finally, the score of the three dimensions was summed up and averaged to obtain the whole score of parent–child relationship. The whole Cronbach’s α is 0.805.

### Analytic Approach

Stata16.0 was used for descriptive statistical analysis and correlation analysis. In order to further investigate the relationship between physical exercise and personality traits, three models were constructed, and stratified regressions were conducted in each model. The dependent variables of model 1, model 2, and model 3 are neuroticism, conscientiousness, and agreeableness, respectively. In step 1, the study added variables at multiple levels, including the items related to the individual, the family and the school, and then included physical exercise in step 2. Finally, SPSS PROCESS3.3 (Hayes, 2012) was used to test the mediating effect of peer relationship and the moderating effect of parent–child relationship between physical exercise and adolescents’ neuroticism, conscientiousness, and agreeableness.

## Results

### Descriptive Analysis and Correlation Analysis

Table 1 summarizes the results of descriptive statistics of the variables. In terms of personal characteristics, male adolescents accounted for 52.2% and female adolescents accounted for 41.8%; rural adolescents accounted for 47.6% and urban adolescents accounted for 52.4%; 74.7% of adolescents believed that their health was in a good condition; the average cognitive ability of adolescents was 22.956, showing a high overall cognitive level; 78.9% of families were in a good financial condition. In terms of school characteristics, 25.9% of adolescents were registered in schools in central urban areas, and 39.8% in urban-rural areas. From the descriptive statistical results of the sample survey, the data were relatively balanced in terms of gender, urban and rural areas, health status, school location, and other aspects.

In Table 2, Step 1 has shown the results of rank-order correlations of three personality domains between the baseline survey and the first follow-up. Spearman correlation results have shown that neuroticism, conscientiousness, and agreeableness were significantly positively correlated in the baseline survey and the follow-up survey. However, from the perspective of mutuality intensity, most of the correlation coefficients are less than 0.4, indicating that the three dimensions of personality traits are weakly correlated. Step 2 has shown the results of the correlation analysis between independent variables, dependent variables, mediating variables, and moderating variables. The results have shown that physical exercise was positively correlated with peer relations, parent–child relationship, and the dimensions of personality traits, including neuroticism, conscientiousness, and agreeableness. Neuroticism was positively correlated with conscientiousness, agreeableness, peer relations, and parent–child relationship. Conscientiousness was positively correlated with agreeableness, peer relations, and parent–child relationship. Agreeableness was positively correlated with peer relations and parent–child relationship. Peer relationship was positively correlated with parent–child relationship.

**TABLE 2:** Correlations of variables (*N* = 9,284).

	1	2	3	4	5	6
**Step 1 Spearman correlation**						
1. Neuroticism^a^	–					
2. Neuroticism^b^	0.42** [0.40, 0.43]	–				
3. Conscientiousness^a^	0.19** [0.17, 0.21]	0.10** [0.07, 0.11]	–			
4. Conscientiousness^b^	0.15** [0.13, 0.17]	0.19** [0.16, 0.21]	0.28** [0.26, 0.30]	–		
5. Agreeableness^a^	0.28** [0.26,0.30]	0.17** [0.15,0.20]	0.27** [0.15, 0.19]	0.24** [0.22, 0.26]	–	
6. Agreeableness^b^	0.22** [0.19, 0.24]	0.27** [0.26, 0.29]	0.16** [0.14, 0.19]	0.34** [0.32, 0.36]	0.43** [0.41, 0.45]	–
**Step 2 Pearson correlation**						
1. Physical exercise	–					
2. Neuroticism^c^	0.07** [0.05, 0.10]	–				
3. Conscientiousness^c^	0.03** [0.01, 0.05]	0.16** [0.13, 0.18]	–			
4. Agreeableness^c^	0.16** [0.14, 0.18]	0.25** [0.23, 0.28]	0.22** [0.20, 0.24]	–		
5. Peer relationship	0.07** [0.04, 0.10]	0.19** [0.15, 0.20]	0.20** [0.18, 0.23]	0.26** [0.24, 0.28]	–	
6. Parent-child relationship	0.22** [0.20, 0.24]	0.23** [0.21, 0.25]	0.12** [0.10, 0.14]	0.36** [0.34, 0.38]	0.21** [0.17, 0.22]	–

**Correlations are significant at the 0.001 level (two-tailed); [,] is the 95% confidence interval. ^a^Baseline survey data. ^b^The follow-up survey data. ^c^The difference between the two periods (Δt).

### Regression Results

Table 3 summarizes the results of the stratified regression of physical exercise on the personality traits of adolescents. In Model 1, variables of three aspects, namely, individuals, families, and schools at multiple levels have been analyzed in the regression equation in Step 1, and then physical exercise was included in the regression equation in Step 2. Both regression equations were statistically significant. Comparing the R^2^ of the two regression models revealed that the explanatory coefficient of the regression equation increased by 0.9% with the inclusion of physical exercise. The regression results of Step 2 indicated the significant positive effect of physical exercise on the development of neuroticism in adolescents.

**TABLE 3:** Regression results of physical exercise and adolescent personality development (*N* = 9,284).

Variable	Step 1			Step 2		
	β	*t*	95% CI	β	*t*	95% CI
**Model 1 Dependent variable: neuroticism**						
Gender	0.02	−1.36	[−0.08, −0.02]	0.02	−1.11	[−0.08, −0.02]
National	0.02	1.42	[−0.02, 0.14]	0.02	1.29	[−0.03, 0.14]
Registered residence	0.04	2.27**	[0.01, 0.11]	0.04	2.25*	[0.09, 0.11]
Cognitive ability	0.08	5.59***	[0.05, 0.11]	0.08	5.53***	[0.05, 0.11]
Health	0.19	13.58***	[0.28, 0.37]	0.19	13.47***	[0.27, 0.37]
One child	0.02	1.25	[−0.02, 0.08]	0.02	1.22	[−0.02, 0.08]
Parental education level	−0.01	−0.80	[−0.04, 0.02]	−0.01	−0.83	[−0.04, 0.02]
Family economic	0.05	3.27***	[0.05, 0.16]	0.05	3.34***	[0.05, 0.16]
Family cultural capital	0.05	3.17***	[0.01, 0.06]	0.05	2.88**	[0.05, 0.16]
School infrastructure	−0.03	−1.71	[−0.11, 0.01]	−0.02	−1.64	[−0.11, 0.01]
School type	0.01	0.66	[−0.09, 0.11]	0.01	0.60	[−0.09, 0.10]
School quality (above average)	0.02	0.97	[−0.04, 0.08]	0.02	0.93	[−0.04, 0.08]
School quality (the best)	0.03	1.60	[−0.03, 0.13]	0.03	1.52	[−0.03, 0.13]
School location (a combination of urban and rural areas)	0.01	0.358	[−0.04, 0.08]	0.01	0.21	[−0.05, 0.08]
School location (city center)	0.05	2.41*	[0.02, 0.15]	0.04	2.32*	[0.02, 0.15]
Physical excise				0.04	2.40*	[0.02, 0.21]
Constant		15.22***	[1.22, 1.58]		15.18***	[1.21, 1.58]

Step 1: R^2^ = 0.070; ΔR^2^ = 0.067; *F* = 20.54; *p* < 0.001 Step 2: R^2^ = 0.061; ΔR^2^ = 0.059; *F* = 19.63; *p* < 0.001						

**Model 2 Dependent variable: conscientiousness**						
Gender	−0.10	−5.47***	[−0.14, −0.07]	−0.08	−5.80***	[−0.15, 0.07]
National	0.01	0.64	[−0.05, 0.09]	0.01	0.46	[−0.06, 0.08]
Registered residence	0.02	0.91	[−0.03, 0.07]	0.01	0.88	[−0.03, 0.06]
Cognitive ability	0.06	3.94***	[0.03, 0.07]	0.06	3.86***	[0.02, 0.07]
Health	0.04	2.87**	[0.02, 0.10]	0.04	2.72**	[0.02, 0.09]
One child	−0.01	−0.12	[−0.05, 0.03]	−0.01	−0.15	[−0.06, 0.03]
Parental education level	0.02	1.04	[−0.02, 0.03]	0.02	0.99	[−0.02, 0.03]
Family economic	0.05	3.06***	[0.03, 0.12]	0.05	2.97**	[0.03, 0.9]
Family cultural capital	0.09	5.55***	[0.04, 0.07]	0.09	5.12***	[0.03, 0.07]
School infrastructure	−0.01	−0.13	[−0.06, 0.04]	0.01	−0.03	[−0.05, 0.05]
School type	0.02	1.46	[−0.02, 0.14]	0.02	1.359	[0.01, 0.10]
School quality (above average)	0.02	1.03	[−0.03, 0.08]	0.02	0.973	[−0.04, 0.08]
School quality (the best)	0.06	2.77**	[0.03, 0.17]	0.05	2.64**	[0.03, 0.16]
School location (a combination of urban and rural areas)	0.01	0.43	[−0.04, 0.07]	0.01	0.21	[−0.04, 0.06]
School location (city center)	−0.01	−0.62	[−0.07, 0.03]	−0.02	−0.75	[0.07, 0.03]
Physical excise				0.05	3.55***	[0.02, 0.21]
Constant		11.78***	[0.77, 1.08]		11.73***	[0.77, 1.07]

Step 1: R^2^ = 0.053; ΔR^2^ = 0.050; *F* = 10.69; *p* < 0.001 Step 2: R^2^ = 0.056; ΔR^2^ = 0.053; *F* = 10.83; *p* < 0.001						

**Model 3 Dependent variable: agreeableness**						
Gender	−0.08	−5.77***	[−0.14, −0.07]	−0.09	−6.57***	[−0.15, −0.08]
National	0.10	6.88***	[0.16, 0.29]	0.09	6.49***	[0.15, 0.28]
Registered residence	0.01	0.30	[−0.03, 0.06]	0.01	0.25	[−0.03, 0.06]
Cognitive ability	0.13	8.73***	[0.01, 0.05]	0.12	8.59***	[0.01, 0.05]
Health	0.11	8.43***	[0.09, 0.16]	0.11	8.13***	[0.09, 0.16]
One child	0.07	4.26***	[0.02, 0.11]	0.06	4.20***	[0.02, 0.10]
Parental education level	0.04	2.19*	[0.01, 0.06]	0.03	2.09*	[0.01, 0.06]
Family economic	0.02	1.67	[−0.01, 0.08]	0.03	1.89	[−0.01, 0.09]
Family cultural capital	0.17	10.50***	[0.05, 0.09]	0.15	9.61***	[0.04, 0.08]
School infrastructure	0.07	5.08***	[0.02, 0.12]	0.07	4.88***	[0.02, 0.12]
School type	0.02	1.42	[0.02, 0.18]	0.02	1.21	[0.02, 0.17]
School quality (above average)	0.07	3.93***	[0.03, 0.13]	0.07	3.84***	[0.03, 0.12]
School quality (the best)	0.15	7.71***	[0.13, 0.25]	0.14	7.48***	[0.12, 0.25]
School location (a combination of urban and rural areas)	0.06	3.44***	[0.07, 0.17]	0.05	2.97**	[0.01, 0.11]
School location (city center)	0.06	3.48***	[0.11, 0.40]	0.06	3.20***	[0.06, 0.16]
Physical excise				0.11	8.08***	[0.21, 0.37]
Constant		3.84***	[0.13, 0.42]		3.51***	[0.11, 0.40]

Step 1: R^2^ = 0.139; ΔR^2^ = 0.136; *F* = 50.25; *p* < 0.001 Step 2: R^2^ = 0.136; ΔR^2^ = 0.133; *F* = 50.23; *p* < 0.001						

**Model 4 Dependent variable: personality traits**						
Gender	−0.06	−4.77***	[−0.09, −0.08]	−0.07	−5.40***	[−0.10, −0.04]
National	0.06	4.35***	[0.06, 0.14]	0.06	4.05***	[0.05, 0.14]
Registered residence	0.03	1.85	[−0.01, 0.06]	0.03	1.84	[−0.01, 0.06]
Cognitive ability	0.14	10.04***	[0.06, 0.10]	0.14	9.97***	[0.06, 0.09]
Health	0.18	13.58***	[0.15, 0.20]	0.18	13.38***	[0.15, 0.20]
One child	0.04	2.35**	[0.01, 0.06]	0.03	2.28*	[0.01, 0.06]
Parental education level	0.02	1.12	[−0.01, 0.03]	0.02	1.06	[−0.01, 0.03]
Family economic	0.02	1.46	[−0.01, 0.06]	0.02	1.63	[−0.01, 0.06]
Family cultural capital	0.15	9.54***	[0.04, 0.07]	0.14	8.80***	[0.04, 0.07]
School infrastructure	0.05	3.51***	[0.03, 0.10]	0.05	3.36***	[0.02, 0.09]
School type	0.03	2.08*	[0.01, 0.11]	0.03	1.96*	[0.01, 0.11]
School quality (above average)	0.05	2.69**	[0.01, 0.08]	0.05	2.62**	[0.01, 0.08]
School quality (the best)	0.11	6.10***	[0.10, 0.18]	0.11	5.92***	[0.10, 0.18]
School location (a combination of urban and rural areas)	0.04	2.38*	[0.01, 0.08]	0.03	2.03*	[0.01, 0.07]
School location (city center)	0.06	3.24***	[0.02, 0.08]	0.05	3.05**	[0.02, 0.09]
Physical excise				0.09	6.61***	[0.12, 0.23]
Constant		16.61***	[0.77, 0.98]		16.53***	[0.76, 0.97]

Step 1: R^2^ = 0.128; ΔR^2^ = 0.126; *F* = 52.70; *p* < 0.001 Step 2: R^2^ = 0.150; ΔR^2^ = 0.147; *F* = 53.55; *p* < 0.001						

*p < 0.05, **p < 0.01, ***p < 0.001.

In Model 2, the study has adopted the stratified regression to test the impact of physical exercise on adolescent conscientiousness. The regression results of the two regression equations showed that the regression equation was statistically significant. By comparing the R^2^ of the two regression models, it was found that the explanatory coefficient of the regression equation increased by 0.3% with the inclusion of physical exercise. The Step 2 regression model showed that physical exercise had a significant positive effect on adolescents’ conscientiousness.

In Model 3, the results of the two regression equations showed that the regression equation was statistically significant. Comparing the R^2^ of the two regression models revealed that the explanatory coefficient of the regression equation increased by 1.1% with the inclusion of physical exercise. The Step 2 regression model showed that physical exercise has a significant positive effect on adolescents’ agreeableness.

According to the regression results of model 4, it is clear that physical exercise has a robust effect on the development of adolescents’ personality traits.

### Moderated Mediation Analysis

Table 4 summarizes the results of the moderated mediation analysis. In Model 1 (shown in Table 4), the results showed that peer relationship played a mediating effect in the effect of physical exercise on adolescent neuroticism. However, the interaction term between physical exercise and parent–child relationship, peer relationship, and parent–child relationship was not significant, which indicated that the parent–child relationship did not have a moderating effect.

**TABLE 4:** Standardized coefficients for the moderated mediation model.

Variable	Coeff	SE	*t*	*P*	LLCI	ULCI
**Model 1 Dependent variable: neuroticism**						
X(Physical exercise)	0.02	0.01	2.53	*P* < 0.01	0.01	0.04
M(Peer relationship)	0.10	0.01	11.58	*P* < 0.001	0.11	0.16
W(Parent-child relationship)	0.12	0.02	18.09	*P* < 0.001	0.34	0.43
M*W	0.02	0.03	1.23	*P* > 0.05	–0.02	0.05
X*W	0.03	0.02	1.41	*P* > 0.05	–0.02	0.05
Constant	0.02	0.01	2.83	*P* < 0.01	0.04	0.16
R^2^ = 0.07; *F* = 148.10; *P* < 0.001; Cohen’s f^2^ 0.75						
**Model 2 Dependent variable: conscientiousness**						
X(Physical exercise)	0.04	0.01	2.86	*P* < 0.01	0.01	0.07
M(Peer relationship)	0.16	0.02	14.84	*P* < 0.001	0.14	0.18
W(Parent-child relationship)	0.13	0.01	11.78	*P* < 0.001	0.11	0.15
M*W	0.03	0.02	2.07	*P* < 0.05	0.02	0.07
X*W	–0.01	0.01	–0.30	*P* > 0.05	–0.03	0.02
Constant	0.02	0.01	2.63	*P* < 0.01	0.03	0.16
R^2^ = 0.08; *F* = 103.92; *P* < 0.001; Cohen’s f^2^ 0.64						
**Model 3 Dependent variable: agreeableness**						
X(Physical exercise)	0.11	0.01	7.81	*P* < 0.001	0.08	0.14
M(Peer relationship)	0.13	0.03	8.83	*P* < 0.001	0.16	0.22
W(Parent-child relationship)	0.13	0.01	8.05	*P* < 0.001	0.15	0.21
M*W	0.12	0.05	2.37	*P* < 0.01	0.02	0.22
X*W	0.12	0.06	2.03	*P* < 0.05	0.02	0.06
Constant	0.02	0.01	2.34	*P* < 0.05	0.06	0.16
R^2^ = 0.10; *F* = 196.54; *P* < 0.001; Cohen’s f^2^ 0.11						

**FIGURE 2:**
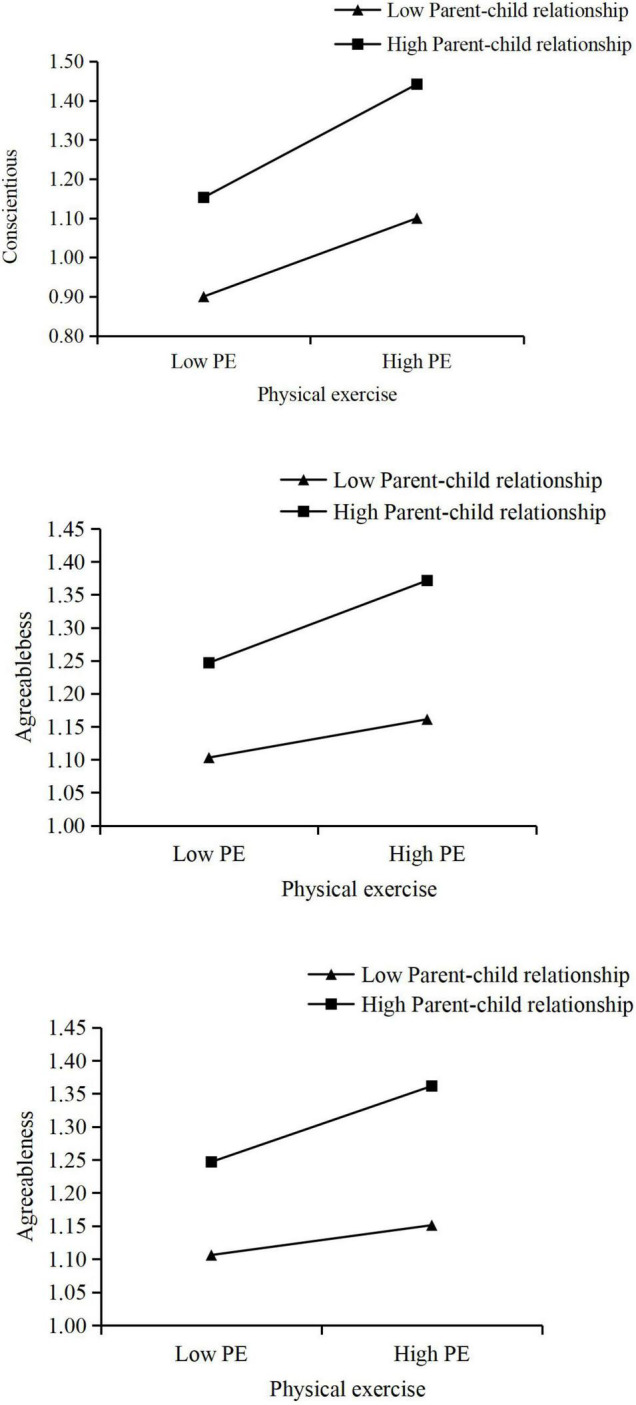
The simple slope indicating the moderation effects. PR, peer relationship; PE, physical exercise.

In Model 2 (shown in Table 4), the results showed that the peer relationship had a mediating effect on the impact of physical exercise on adolescent conscientiousness. In terms of moderating effect, the interaction term between peer relationship and parent–child relationship was significant, which suggested that the parent–child relationship moderated the effect of peer relationship on adolescents’ conscientiousness. The simple slope plot of the moderating effect showed that physical exercise positively predicted adolescents’ conscientiousness at high levels of parent–child relationship (shown in Figure 2). The moderating effect of high levels of parent–child relationship on physical exercise and adolescent conscientiousness was more significant than the effect of low levels of parent–child relationship. However, the interaction term between physical exercise and parent–child relationship was not significant, suggesting that the parent–child relationship did not have a moderating effect on the “physical exercise→ conscientiousness” (Mo1) pathway.

In Model 3 (shown in Table 4), the results showed that peer relationship had a mediating effect on the impact of physical exercise on adolescent agreeableness. In terms of moderating effects, the interaction between peer relations and parent–child relations was significant, indicating that the parent–child relationship moderated the effect of peer relations on adolescents’ agreeableness. The simple slope plot of the moderating effect showed that peer relationships positively predicted adolescents’ agreeableness at high levels of parent–child relationships (shown in Figure 2). The high-level parent–child relationship had a more significant regulatory effect on peer relationship and adolescents’ agreeableness than the low-level parent–child relationship. The moderating effect of high levels of parent–child relationships on peer relationships and adolescent agreeableness was more significant than the effect of low levels of parent–child relationships. The interaction between physical exercise and parent–child relationship was significant, indicating that the parent–child relationship moderated the effect of physical exercise on adolescent agreeableness (shown in Table 4). The simple slope plot of the moderating effect showed that physical exercise positively predicted the level of adolescent agreeableness at high levels of parent–child relationship (shown in Figure 2). The moderating effect of high levels of parent–child relationship on physical exercise and adolescent agreeableness was more significant than the effect of low levels of parent–child relationship.

## Discussion

Based on the EST, this study has constructed a moderated mediation model with peer relationship as the mediating variable and parent–child relationship as the moderating variable, and discussed the influence of physical exercise on adolescents’ personality traits as well as the joint effect of peer and family microsystems on the development of adolescent personality traits.

The results have shown that physical exercise could significantly predict adolescent neuroticism, conscientiousness, and agreeableness, which are consistent with the existing studies. In terms of influence effect, the research results have shown that physical exercise has a 4% influence effect on adolescent neuroticism, a 5% influence effect on adolescent conscientiousness, and an 11% influence effect on adolescent agreeableness. The results are consistent with the existing research (Allen and Laborde, 2014), but have the subtle differences of the specific value of influence effect. For example, research by Stephan has issued that the effect of physical exercise on adolescent neuroticism is 2%, the effect on conscientiousness is 4%, and the effect on appropriate agreeableness is 4% (Stephan et al., 2014b). Comparing the two studies, this study has shown a higher impact. Specifically, physical exercise could promote the release of beta-endorphins (Legey et al., 2017) and brain-derived neurotrophic factor (Donati et al., 2021), and help them maintain positive emotions and promote the stability of neuroticism. In addition, for adolescents, active participation in physical exercise could alleviate academic stress and help them release negative emotions and maintain mental health (Motl et al., 2004; Wiles et al., 2008; Jewett et al., 2014). In terms of adolescents’ conscientiousness, it is mainly reflected that when taking physical exercise, it needs to overcome difficulties and enable students to experience the successful experience of completing goals and conscientiousness (de Bruijn et al., 2009; Eime et al., 2013). Long-term participation in physical exercise not only requires adolescents to have a high degree of self-discipline, but also may increase adolescents’ conscientiousness (Gallagher et al., 2013). In terms of adolescents’ agreeableness, actively participating in physical exercise could effectively improve adolescents’ cognitive flexibility (Bhattacharyya et al., 2022) and social communication ability (Stephan et al., 2014a), promote adolescents’ emotional acquisition, maintain adolescents’ prosocial behavior, and then promote the development of adolescents’ agreeableness (Stephan et al., 2014b).

The results of mediation effect show that peer relationship plays the mediating role between physical exercise and the development of personality traits such as neuroticism, conscientiousness, and agreeableness. Physical exercise could not only directly predict the development of adolescent’s personality traits, but also significantly predict the development of adolescent personality traits through peer relationships. In adolescence, peer relationship has gradually become an important scene in adolescents’ development. In the process of physical exercise, adolescents could enhance peer relationship, expand the scope of interpersonal communication, and develop the level of personality traits under the influence of peer effect through physical exercise (Smith, 2003). Meanwhile, participating in physical exercise could make young people feel better about peer acceptance and friendship connection, and show more positive behaviors and attitudes in the process of sports participation, thereby enhancing the sense of sports acquisition, satisfying their social needs, and emotional communication, and developing personality traits (Bandura, 1989; Eagleton et al., 2007; Zinchenko et al., 2018; Allen et al., 2020).

The results of the moderating effect show that the interaction of peer relationship and parent–child relationship jointly affects the development of adolescent’ conscientiousness in the impact of physical exercise on adolescent conscientiousness. In terms of the impact of physical exercise on adolescent’ agreeableness, the interaction between physical exercise and parent–child relationship and the interaction between parent–child relationship and peer relationship combined to influence the development of adolescent agreeableness. Based on the theory of human ecosystem, individual behavior is affected by the interaction with the environment. In the three-layer model of micro, medium, and macro, family and peers, as the main components of the microsystem, play an irreplaceable role in the growth and development of adolescents (Overton, 2013a,b). Parent–child relationship plays an important role in the construction of adolescent self-cognition. A high level of parent–child relationship would make adolescents form a safe world view (Greenberg, 1999), more inclined to get along with others and participate in physical exercise with a more positive attitude and behavior, reduce the occurrence of problem behaviors (Hutteman et al., 2014), and promote the development of adolescent personality traits to a greater extent, such as neuroticism, extroversion, and agreeableness (McCarthy, 2007; Clark et al., 2018). Meanwhile, a good parent–child relationship could effectively intervene and weaken the influence of negative peer behaviors on adolescents. Under the influence of good parent–child relationship and peer relationship, adolescents tend to show positive social behaviors in the process of communication with others, promote the acquisition of social emotion, and make teenagers have more affinity (Winefield et al., 2015; Raufelder et al., 2021).

## Conclusion

This study has investigated the effect of physical exercise on the development of adolescents’ personality traits, and the effect of mechanism of peer relationship and parent–child relationship on physical exercise and personality traits. It has been found that physical exercise has a significant positive predictive effect on the development of personality traits such as neuroticism, conscientiousness, and agreeableness. Physical exercise could affect adolescents’ neuroticism, conscientiousness, and agreeableness through peer relationship. In addition, parent–child relationships show differential moderating effects in different models. In the influential model of physical exercise on adolescents’ neuroticism, the moderating effect of parent–child relationship is not significant. In the influential model of physical exercise on adolescents’ conscientiousness, the parent–child relationship only exerts the direct effect of peer relationship on adolescents’ conscientiousness. In the influential model of physical exercise on adolescents’ agreeableness, the parent–child relationship regulates the direct effect of physical exercise on adolescents’ agreeableness and the direct effect of peer relationship with adolescent agreeableness.

This study would give the enlightenment and guidance for intervention and development of adolescents’ personality traits based on revealing the micro-level influencing mechanism of family and peer system. Moreover, the study includes the influencing factors of individuals, families, and schools in the regression analysis, which makes the model more explanatory, and provides important theoretical support and practical guidance for further scientific formulation of adolescent’s physical exercise plan and promoting the development of adolescent’s personality traits. Also, the study has fully considered the typical stability property of personality traits, and made the findings more convincing by using the differences in personality traits before and after the two periods of follow-up survey data as the dependent variable.

There are following limitations in the study. First, due to the particularity of data sources, the measurement of physical exercise only uses the exercise duration as the measurement index, and in the further research, the dimensions such as exercise intensity or events could be added. Second, the measurement of neuroticism in personality traits has some limitation. Although “International personality item pool” and “NEO personality inventory revised” include emotions such as anxiety and anger, it is insufficient only to use the emotional changes in the last 7 days to test neuroticism (Costa and Mccrae, 1992; Goldberg et al., 2006). Third, due to the data source of CEPS, the study could not completely obtain all the variables in the big five personality traits. Only the personality of neuroticism, conscientiousness, and agreeableness has been identified. Furthermore, the survey of parent–child relationship mainly comes from the subjective reports of adolescents, while the reports from the parents of adolescents have not been collected. A more objective and multidimensional measurement could be adopted in the follow-up study.

## Data Availability Statement

Publicly available datasets were analyzed in this study. This data can be found here: http://ceps.ruc.edu.cn/.

## Ethics Statement

Ethical review and approval was not required for the study on human participants in accordance with the local legislation and institutional requirements. Written informed consent from the patients/ participants or patients/participants legal guardian/next of kin was not required to participate in this study in accordance with the national legislation and the institutional requirements.

## Author Contributions

YL was mainly responsible for topic design, analysis, manuscript writing, and study revision. XC was mainly responsible for data analysis and manuscript writing. WC was mainly responsible for revision, proofreading, and supervision of studies. XP was mainly responsible for manuscript writing. All authors contributed to the article and approved the submitted version.

## Conflict of Interest

The authors declare that the research was conducted in the absence of any commercial or financial relationships that could be construed as a potential conflict of interest.

## Publisher’s Note

All claims expressed in this article are solely those of the authors and do not necessarily represent those of their affiliated organizations, or those of the publisher, the editors and the reviewers. Any product that may be evaluated in this article, or claim that may be made by its manufacturer, is not guaranteed or endorsed by the publisher.
